# Protective Effect of *Lactobacillus plantarum* R2 and *Lactobacillus sakei* B2 on Low-Salt Sliced Sausages Stored at 5 °C

**DOI:** 10.3390/foods13233960

**Published:** 2024-12-08

**Authors:** Huiting Luo, Mei Xu, Peijun Li

**Affiliations:** School of Food and Biological Engineering, Hefei University of Technology, Hefei 230009, China; luoht@hfut.edu.cn (H.L.); m13866107822_1@163.com (M.X.)

**Keywords:** protective culture, low sodium, cooked sausage, microbial community, oxidation

## Abstract

This study aimed to investigate the protective effects of inoculating *Lactobacillus plantarum* R2 and *Lactobacillus sakei* B2 on low-salt sliced chicken sausages during storage at 5 °C. The results demonstrated that *L. plantarum* R2 inhibited the growth of *Pseudomonas fluorescens* (*p* < 0.05). The results of the high-throughput sequencing indicated that the chicken sausage inoculated with *L. plantarum* R2 improved the microbiological quality of the sample. The levels of thiobarbituric acid reactive substances and carbonyl content of the sausages treated with *L. plantarum* R2 and *L. sakei* B2 were lower than those of the control (*p* < 0.05). *L. plantarum* R2 exhibited a higher antioxidant activity compared to that of *L. sakei* B2. Therefore, *L. plantarum* R2 was found to have the potential to improve physicochemical properties, organoleptic characteristics, and food safety of low-salt sliced cooked chicken sausages.

## 1. Introduction

The main component of salt is sodium chloride, which can not only improve the texture of meat products, but also give food a special flavor, and suppress the growth of pathogenic and spoilage microorganisms in meat products, extending their shelf life. However, excessive sodium intake can cause a marked elevation in blood pressure, heart disease, and stroke disease risk among salt-sensitive individuals [1,2,3]. Meat products represent the majority of the sodium ingested in diet [4]. With the demand for healthy concepts, it has become a tendency to choose low-salt meat products to reduce the content of sodium by consumers [5]. The World Health Organization (WHO) advocates striving to achieve a 30% reduction in NaCl intake by 2025 [6]. The development of low-salt meat products has gradually become a research hotspot in the meat industry. A prevalent approach for salt reduction is to substitute NaCl with alternative chlorides. The partial replacement of NaCl with KCl seems to be the optimal choice for decreasing the sodium content in meat products. [7,8,9], and it can also contribute to the reduction in the risk of hypertension and cardiovascular diseases [10]. It has been reported that substituting NaCl with KCl is tolerable to consumers provided that the substitution does not exceed 40% [11].

Therefore, reducing the sodium content will not only affect the sensory characteristics such as flavor and texture, but more importantly, it will reduce the microbial stability of meat products [12,13,14]. Aaslyng et al. reported that the microbial community in the product might change when the salt content is reduced [14]. Understanding microbial behavior during storage time is one of the key aspects when considering the safety of low-sodium food [15]. However, there were few studies focused on microbial behavior and food safety in cooked meat products with lower sodium content. Sodium reduction in meat must be combined with other technologies to inhibit the multiplication of spoilage and pathogenic microorganisms and ensure storage stability [16,17]. High-pressure processing (HPP) and ultrasonic technology can be used to extend the shelf life of low-salt meat products, which may cause quality deterioration of meat products [18,19]. New methods for low-salt meat preservation need to be developed.

More natural interventions have been widely studied by the food processing industry. Biological protection technology is a new green quality control technology, which has been applied to meat preservation, including the application of protective cultures. Bioprotective bacteria, as a safe, harmless, and efficient preservative, can effectively inhibit the growth of harmful microorganisms. Lactic acid bacteria (LABs) are the microbial groups most commonly used, for they possess a protracted history of secure utilization and are generally recognized as safe (GRAS) [20,21]. Some LAB species have been reported to play a protective role against pathogenic and spoilage organisms in meat products. Castellano et al. inoculated *Lactobacillus curvatus* CRL705 on the surface of vacuum-packed beef and found that it inhibited the growth of *Brochothrix thermosphacta* and spoilage LABs and extended the shelf life of the product [22]. Slima et al. inoculated *Lactobacillus plantarum* TN8 and *Lactobacillus acidilactici* MA 18/5M into beef sausages stored at 4 °C, and the results showed that the inoculated groups could control the growth of *Enterobacteriaceae* in beef sausages and reduce the cooking loss of products [23]. Comi et al. inoculated the mixed bioprotective bacteria into the beef hamburger with modified atmosphere packaging, which found that the inoculated bacteria could significantly improve the physicochemical quality and microbial community structure of the beef hamburger during storage and prolong the shelf life [24]. Hu et al. inoculated *Lactobacillus sakei* B2 onto vacuum-packed smoked ham slices and found that *L. sake* B2 can prolong the shelf life of ham and inhibit the growth of dominant spoilage bacteria [25]. Thus, the use of LABs with a wide range of antimicrobial activities can enhance meat quality and inhibit the growth of certain spoilage bacteria in order to reduce microbial contamination, thus improving the storage stability aspects of meat products. However, considering the difference in microflora, the effects of protective cultures on the quality of low-salt meat products during storage have not been reported.

The study aimed to evaluate the effect of *L. plantarum* R2 and *L. sakei* B2 on the quality of low-salt sausage stored at 5° C. Culture-dependent and culture-independent methods were used to reveal the bacterial communities during storage.

## 2. Materials and Methods

### 2.1. Bacterial Strains and Growth Conditions

Two bacterial strains were selected for the study. *L. plantarum* R2 was derived from dry sausage, which is a naturally fermented Chinese-style meat product. *L. sakei* B2, being a commercial protective culture, was procured from Chr. Hansen (Hørsholm, Denmark). De Man–Rogosa–Sharpe (MRS, Guangdong Huankai Microbial Sci. & Tech. Co, Ltd., Guangzhou, China) broth served as the media for cultivating the two strains at 37 °C for 24 h.

### 2.2. Preparation of Sausages

Frozen chicken breast and pork backfat were bought from local markets and were then thawed at 4 °C. The cooked chicken sausages were manufactured as described in Table 1 according to Liu et al. [26]. All the sausages were made with a reduced NaCl content (1.75 g/100 g meat), with a 30% substitution of NaCl by KCl (0.75 g/100 g meat). Chicken meat and pork backfat pre-assigned at a ratio of 3:1 (*w*/*w*) were ground through a meat grinder (Shanghai Shuangdie Kitchenware Co., Ltd., Shanghai, China) assembling a 5 mm plate, then mixed with other ingredients, including polyphosphates, sugar, ice water, NaCl, and KCl, for 3 min. The meat batter was cured at 4 °C for 16 h and stuffed tightly into plastic casings of 3.2 cm in diameter. The chicken sausage was prepared with 8–10 cm in length and then heated at 80 °C for 20 min. After heating, the cooked chicken sausages were sliced into 0.5 cm thick pieces within a sterile setting. The sausage slices were inoculated with either *L. plantarum* R2 or *L. sakei* B2 at a final level of 10^7^ CFU/g, while the control sample was formulated without any inoculation. All of the samples were vacuum-packaged in a sterile homogeneous bag (Qingdao Haibo Biotechnology Co., Ltd., Qingdao, China) and stored in refrigeration (5 ± 1 °C) and collected at 0, 4, 8, 12, 16, 24, and 32 d during the storage period.

### 2.3. Measurement of Lipid Oxidation

Lipid oxidation was assessed with the 2-thiobarbituric acid method as described by Jayawardan et al. and Devatkal et al. with slight modifications [27,28]. Thiobarbituric acid reactive substances (TBARSs) of the sausage samples were determined during storage time. Briefly, 5.0 g of the sausage sample was homogenized with 50 mL of 10% (*w*/*v*) trichloroacetic acid (TCA, including 0.1% EDTA-Na_2_) at 3000 rpm over ice for 60 s. The resultant mixture was filtrated through double layers of slow-rate quantitative filter paper (No.203, Hangzhou Special Paper Industry Co., Ltd., Hangzhou, China). The filtrate (5 mL) added with 5 mL of 0.02 M 2-thiobarbituric acid was then heated for 40 min in a water bath maintained at 90 °C. After being cooled down to room temperature, the absorbance was measured at 532 nm. 1,1,3,3-tetraethoxypropane was employed as a positive control for constructing the standard curve. The TBARS value was computed as equivalent to malondialdehyde (MDA) mg/100 g meat.

### 2.4. Measurement of Protein Carbonyl Content

The protein carbonyl content was evaluated by the method of Berardo et al. and Xiao et al. [29,30]. A total of 3 g of each sample was added into 30 mL of phosphate buffer (20 mM, pH 6.5, containing 0.6 M NaCl) and homogenized at a high speed (10000 rpm) for 60 s at 4 °C. Precipitation was collected by centrifugation (12,100× *g*, 15 min) from 0.2 mL homogenate with 1 mL 10% TCA (*w*/*v*) aqueous solution at 4 °C, and reacted with 0.5 mL 10 mM 2,4-dinitrophenylhydrazine (DNPH), while one group reacted with 0.5 mL 2 mM HCl was prepared as a control. After shaking treatment for 1 h, the mixture was added with 20% ice-cold trichloroacetic acid (*w*/*v*) to centrifuge at 8000× *g* for 10 min at 4 °C. The protein precipitation was rinsed three times with 1 mL ethanol and ethyl acetate (*v*/*v*) at the rate of 1:1, then dissolved in 6 M guanidine HCl for 15 min at 37 °C. The absorbance of the final supernatant was detected at 280 nm and 370 nm after centrifugated at 8000 rpm for 10 min (4 °C), respectively. The results were presented as (nmol/mg protein) calculated from the absorbance of the samples using the following equation:

(1) $$\frac{C_{protein}}{C_{hydrazine}}=\frac{A_{370}}{\varepsilon_{hydrazone}\times (A_{280}-A_{370}\times 0.43)}\times 10^{6}$$

where ε_hydrazone_ is 22 mM·cm^−1^ and the carbonyl concentrations obtained from the blanks were subtracted from the corresponding treated sample.

### 2.5. pH Measurement

The pH was determined by mixing 5 g of the sample with 45 mL of distilled water. An FE28 pH meter (Mettler Toledo, Columbus, OH, USA) was used for the measurement.

### 2.6. Bacterial Counting

All the samples were subjected to bacterial counts during the storage. In total, 25 g of each sausage sample was homogenized with 225 mL sterile saline solution and blended in a stomacher for 3 min, thus making a 1/10 dilution. Serial 10-fold dilutions were prepared. Total viable counts were enumerated on Plate Count Agar (PCA; Guangdong Huankai Microbial Sci. & Tech. Co, Ltd., Guangzhou, China) and incubated at 37 °C for 48 h. Numbers of *Pseudomonas spp.* were counted on cephaloridine fucidin cetrimide (CFC; Hopebio, Qingdao, China) agar at 25 °C for 48 h. *B. thermosphacta* counts were counted on streptomycin thallous acetate actidione (STAA; Hopebio, Qingdao, China) agar incubated for 48 h at 25 °C. LAB counts were obtained on MRS agar (Guangdong Huankai Microbial Sci. & Tech. Co, Ltd., Guangzhou, China) for 48 h incubation at 37 °C in an anaerobic environment.

### 2.7. High-Throughput Sequencing

#### 2.7.1. DNA Extraction

For each sample, total bacteria were obtained by the methods of Xiao et al. [30]. Briefly, 0.5 g of sausage was added to 1000 μL of CTAB lysate containing lysozyme, and the sample was fully lysed in a 65 °C water bath. After centrifugation (4000× *g*, 30 min), the compound reagent (phenol (pH 8.0)/chloroform/isoamyl alcohol = 25:24:1, *v*/*v*) was blended with the supernatant, and the mixture was centrifuged at 12,000 rpm for 10 min. The aforementioned operation was reiterated once, and the supernatant was pipetted into a 1.5 mL centrifuge tube, shaken with isopropyl alcohol, and then precipitated at −20 °C. DNA sediment was gained through centrifuging at 12,000 rpm for 10 min and washing twice with 75 % ethanol. The DNA sample was dissolved by ddH_2_O, using 1 μL of RNase A to digest the RNA, and finally stored at –20 °C.

#### 2.7.2. PCR

The primers 515F (50-GTGCCAGCMGCCGCGGTAA-30) and 806R (50-GGACTACHVGGGTWTCTAAT-30) were used to amplify the V4 region of the 16S rRNA gene [30]. The PCR reaction system (30 µL) consisted of 15 µL of Phusion^®^ High-Fidelity PCR Master Mix (New England Biolabs, Ipswich, MA, USA), 1.5 µL of each primer (0.2 µM), and 2 µL of DNA under the following thermal cycling conditions: 98 °C for 1 min and 30 cycles of 94 °C for 30 s, 56 °C for 30 s, and 72 °C for 1 min. The reaction was terminated with an extension step of 5 min at 72 °C. The PCR products were verified by electrophoresis on a 2% (*w*/*v*) agarose gel and then stored at −20 °C.

#### 2.7.3. Sequence of 16S rDNA Amplicons

Analysis of the sequence of 16S rDNA amplicons was achieved by using the TruSeq DNA PCR Free Library Preparation Kit (Illumina, San Diego, CA, USA). The library quality was evaluated in the Qubit@ 2.0 Fluorometer (Thermo Fisher Scientific, Waltham, MA, USA) and Agilent Bioanalyzer 2100 system. Finally, the library was sequenced on an Illumina HiSeq platform (Novogene Bioinformatics Technology Co., Ltd., Beijing, China).

### 2.8. Sensory Evaluation

In strict accordance with the relevant standards, the sensory evaluation was conducted via a double-blind test. The panel consisted of 10 panelists, who were trained for 2 w according to the Chinese National Standard GB/T 22210-2008 [31], for adaptation to the specific attributes of the sausages. Color, odor, texture, viscosity, and overall acceptability, which are the most concerning aspects for consumers and also common indicators in meat products, were selected as the evaluation attributes. Among them, the texture and viscosity were assessed through the perception of the panelists’ fingers. All sessions were held at room temperature in a sensory panel room equipped with white fluorescent lighting. The samples were numbered with random digits and served on white plates in random order. The panel members conducted independent evaluations of each sample for the attributes. A glass of about 100 mL of water was provided to gargle for each panelist between samples. All of the attributes were evaluated on a l–9 hedonic scale as described by Sallam et al. and Liu et al. with slight modifications [26,32], in which the scores were grouped in the following intervals: 1–4 poor color, bad smell, poor texture, sticky, very poor acceptability; 4–6 color is normal, a little odor, poor texture, began to produce sticky residue, relatively poor acceptability; 6–9, good texture, no slime, very good acceptability.

### 2.9. Statistical Analysis

Three independent experiments were performed on different days, with each being replicated three times. Different treatments (unadded, *L. plantarum* R2 added, and *L. sakei* B2 added) were included as fixed effects and replicated as random effects. Data were subjected to analysis of variance with the utilization of the Statistix 8.1 software package (Analytical Software, St. Paul, MN, USA). The General Linear Models procedure was used to determine the significance. Significant differences (*p* < 0.05) among means were detected using Tukey’s honest significant difference procedure.

## 3. Results and Discussion

### 3.1. Lipid Oxidation

**Figure 1:**
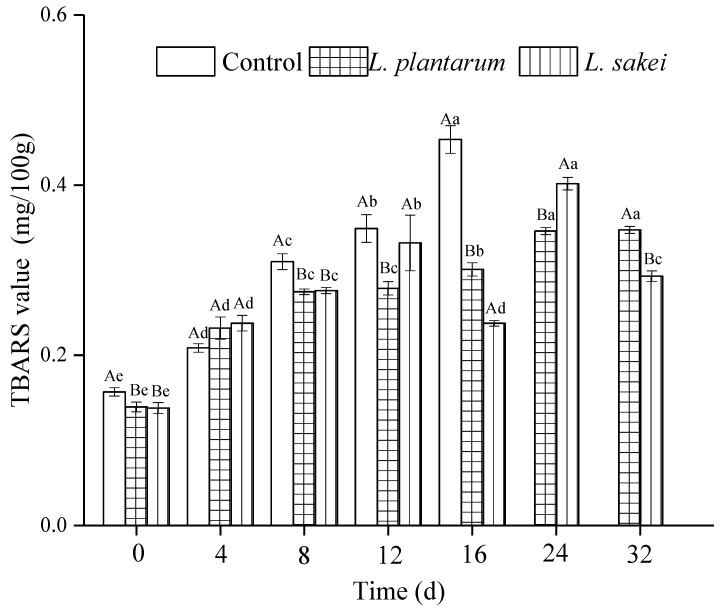
Effect of inoculating *L. plantarum* R2 and *L. sakei* B2 on the TBARS value of low-sodium sliced sausages during refrigerated storage (5 ± 1 °C). Means labeled with different uppercase letters (A, B) represent significant differences between different treatments at the same time (*p* < 0.05), and means labeled with different lowercase letters (a–e) represent significant differences among different times for the same treatment (*p* < 0.05).

TBARS values reflect the level of the byproducts of lipid oxidation, MDA, whose content is typically utilized to signify the state of lipid oxidation. The TBARS values in all the samples increased during storage, as shown in Figure 1. After 8 d of storage, the TBARS values of the sausages treated with either *L. plantarum* R2 or *L. sakei* B2 were lower in comparison to that of the control (*p* < 0.05). The results indicated that *L. plantarum* R2 and *L. sakei* B2 were effective in delaying the fat oxidation of the low-sodium chicken sausages [33,34,35,36]. Moderate lipid oxidation can contribute to the formation of the characteristic flavors of meat products, but excessive lipid oxidation may cause significant deterioration of meat product quality [37]. Pigment protein and Fe^2+^ are the most basic catalysts for fat oxidation of meat products. Pigment protein is deactivated during heating, so Fe^2+^ has a great impact on the lipid oxidation process of meat products. The decreased TBARS values of the inoculated sausages may be due to the apparently antioxidant activity of two bacteria strains. Research studies have shown that some bioprotective bacteria possess significant antioxidant activity due to their free radical scavenging activity and Fe^2+^-chelating ability [38,39]. Previous studies also reported that some microbes had the ability to selectively decompose and utilize carbonyl compounds like MDA, or to further oxidize MDA to carboxylic acid [40,41], which may lead to a reduction in the TBARS values. This mechanism needs further experimental exploration.

Some have the ability to selectively decompose and utilize carbonyl compounds such as MDA.

The relationship between the TBARS value and microbial communities is complex [34]. Thus, *L. plantarum* R2 and *L. sakei* B2 inoculation inhibited the growth of *Enterobacteriaceae, Staphylococcus aureus*, and *Micrococcus* spp. Similar results were found in that an *L. curvatus* strain showed relevant antimicrobial activities against *S. aureus* and *Enterobacteriaceae* in fermented meat [21].

### 3.2. Protein Oxidation

**Figure 2:**
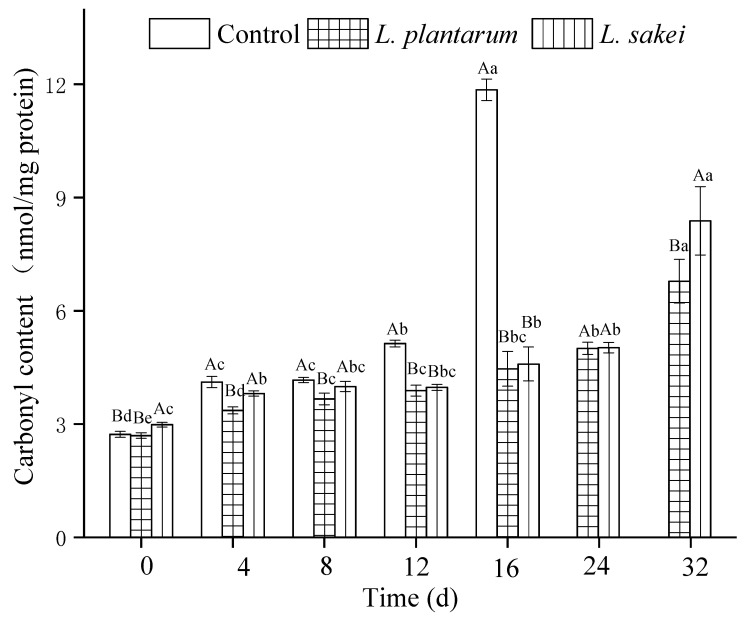
Effect of inoculating *L. plantarum* R2 and *L. sakei* B2 on the carbonyl content of low-sodium sliced sausages during refrigerated storage (5 ± 1 °C). Means labeled with different uppercase letters (A, B) represent significant differences between different treatments at the same time points (*p* < 0.05), and means labeled with different lowercase letters (a–e) represent significant differences among different times for the same treatment (*p* < 0.05).

The protein oxidation levels in the samples were evaluated by the content of carbonyls, which indicates the oxidation progression. The carbonyl content of all the samples increased during refrigerated storage, as shown in Figure 2. At 12 d, the carbonyl content in the control samples was 5.13 mg nmol/mg protein, and it was reduced by 24.3% and 22.6% in the *L. plantarum* and *L. sakei* samples, respectively. In the present study, the samples containing protective cultures exhibited considerable efficiency in preventing the formation of protein carbonyls. Similar findings were documented by Berardo et al. [29], who reported that the protein carbonyl was formed mainly through three ways: metal-catalyzed oxidation, non-enzymatic glycation, and the formation of the adduct with non-protein carbonyl compounds. During metal-catalyzed oxidation, Fe^2+^ stimulates the formation of oxygen radicals from oxygen and H_2_O_2_ through the Fenton reaction [42]. Both protein oxidation and lipid oxidation can be caused by free radical chain reaction, in which oxygen free radicals react with side chain groups of amino acids to form carbonyl compounds. According to Tang et al. and Geet et al. [33,36], *L. plantarum* had Fe^2+^-chelating abilities and various free radical scavenging capacities. The present study further demonstrated the inhibitory effect of some probiotic LABs on protein oxidation. Upon the completion of storage, an obvious difference was observed between the samples inoculated with *L. plantarum* and *L. sakei*. The carbonyl content in the group added with *L. plantarum* was lower than that in the sample inoculated with *L. sakei*. This may be due to the higher antioxidative properties of the *L. plantarum* strain. Thus, the two LAB strains exhibited inhibition of protein oxidation, which was in agreement with the TBARS values (Figure 1). Similar results have also been reported in an earlier study [28].

### 3.3. pH

**Figure 3:**
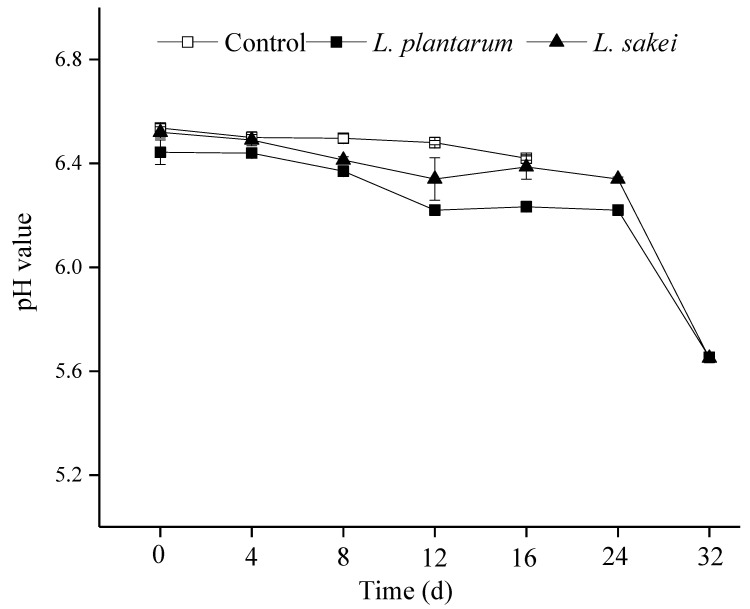
Effect of inoculating *L. plantarum* R2 and *L. sakei* B2 on the pH value of low-sodium sliced sausages during refrigerated storage (5 ± 1 °C).

The pH value represents a crucial parameter in the surveillance of meat quality, which has a large impact on the color, lipid oxidation, and sensory quality of meat products. As shown in Figure 3, during the first 4 days of storage, the pH values of all the samples remained stable at approximately 6.4–6.5. Then, the pH values of the inoculated sausages showed a decrease from day 4 to day 12. The reduction in the pH was probably due to the accumulation of lactic acid produced from the glycometabolism by the LAB strains’ growth [43]. As time progressed, the pH values of the two inoculated samples slightly fluctuated, but not significantly (*p* > 0.05). The pH kept a value of more than 6.2 during the first 24 days. After 24 d of storage, the pH value of the treated groups began to sharply decrease, possibly due to the overgrowth of certain microorganisms in the product (such as LABs and *Leuconostoc mesenteroides*), which decomposed a large amount of carbohydrates in meat products and accumulated excessive lactic acid. These were considered to be unacceptable by the consumers [44].

### 3.4. Microbial Communities

#### 3.4.1. Bacterial Counts

**Figure 4:**
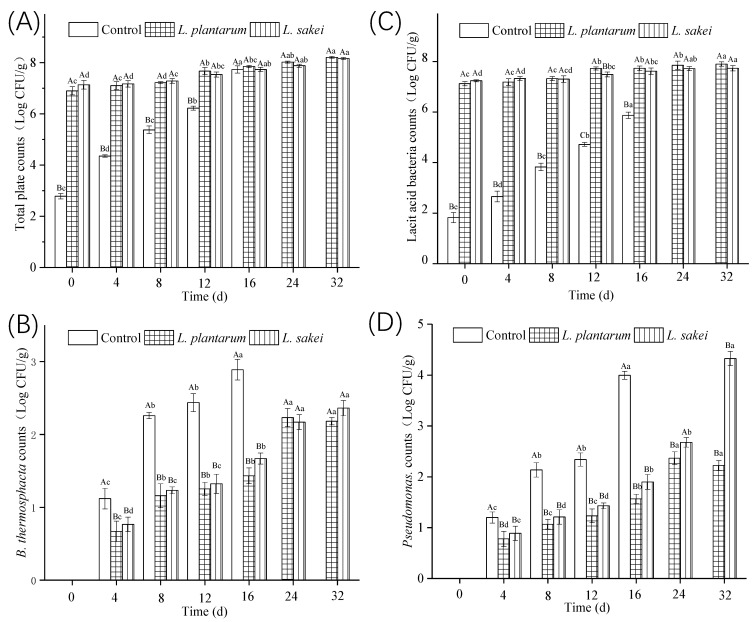
Effect of inoculating *L. plantarum* R2 and *L. sakei* B2 on the counts of total plate count (**A**), LABs (**B**), *B. thermosphacta* (**C**), and *Pseudomonas* spp. (**D**) of low-sodium sliced sausages during refrigerated storage (5 ± 1 °C). Means labeled with different uppercase letters (A–C) represent significant differences between different treatments at the same time points (*p* < 0.05), and means labeled with different lowercase letters (a–e) represent significant differences among different times for the same treatment (*p* < 0.05).

The total plate counts of all the samples exhibited an upward trend during the period of refrigerated storage, as shown in Figure 4A. At the beginning, the total plate count of the control was 2.78 log CFU/g. Then, the count increased to a final concentration of 7.73 log CFU/g after 16d of storage. Kreyenschmidt et al. pointed out that 6 log CFU/g or higher of the total plate count in cooked ham was an indication of spoilage [45]. In this study, it was found that the total plate count of the control was more than 6 log CFU/g at 12 d. Thus, 12 d was considered to be the shelf life for the control during microbial analysis.

The LAB counts in the cooked sausage samples are presented in Figure 4B. The counts of LABs in the samples treated with *L. plantarum* R2 and *L. sakei* B2 were 7.11 log CFU/g and 7.24 CFU/g at 0 d, respectively, which were not different from those of the total plate counts (*p* > 0.05; Figure 4A). Thus, the LABs were dominated in the two inoculated samples at first. During the storage, the bacteria grew slowly and there was no significant difference until 12 d (*p* > 0.05). This was important regarding the sausages because the rapid multiplication of the bacteria would cause a rapid reduction in pH, which would be unacceptable to the consumers. In fact, the pH remained unchanged during the first 24 days of storage (Figure 3).

*B. thermosphacta* is a meat-borne microorganism that is usually responsible for the spoilage of refrigerated meat products under both aerobiosis and anaerobiosis. In the beginning, it was not detected in all the samples (Figure 4C). At 16 d, the *B. thermosphacta* count achieved a maximum of 3.91 log CFU/g in the control group. In the two inoculated samples, the growth of *B. thermosphacta* was effectively inhibited (*p* < 0.05), when compared with that of the control. It was found that there was no difference in the *B. thermosphacta* counts of the two samples, which means that *L. plantarum* R2 and *L. sakei* B2 showed similar antimicrobial effects against *B. thermosphacta*. Similar results have also been reported in meat products by other investigators [46,47].

As reported in other studies, *P. fluorescens* were known to be mainly composed of the initial microflora and to prevail on meat stored at chill temperatures under aerobic storage conditions [14,48]. Statistical analysis showed that *P. fluorescens* were significantly inhibited in all inoculated chicken sausages. Spanu et al. found that the bioprotective culture had an effective ability against *P. fluorescens* growth [49]. However, the inoculum of *L. plantarum* R2 on vacuum-packaged chicken sausage significantly suppressed the growth of *P. fluorescens* when compared to the inoculum of *L. sakei* B2. This may be due to the fact that *L. plantarum* R2 was more effective against *P. fluorescens* than *L. sakei* B2 in chicken sausage. In the control group, almost no *P. fluorescens* was detected at 0d, and *P. fluorescens* counts increased from a starting concentration of 1.21 ± 0.11 log CFU g^−1^ at 4 d to a final concentration of 3.98 ± 0.15 log CFU g^−1^ at 16 d (Figure 4).

#### 3.4.2. High-Throughput Sequencing Analysis

**Figure 5:**
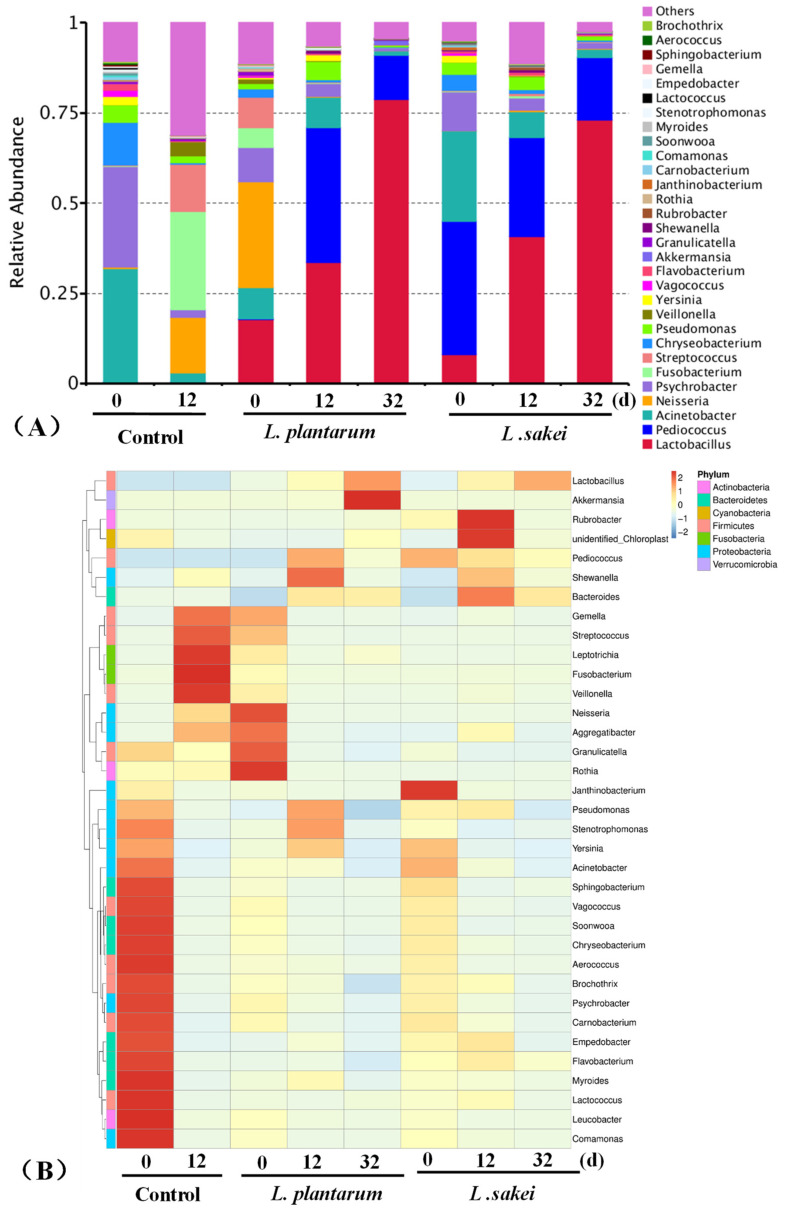
Effect of inoculating *L. plantarum* R2 and *L. sakei* B2 on the bacterial relative abundance (**A**) and a hierarchically clustered heat map (**B**) at the genus level in low-sodium sliced sausages during refrigerated storage (5 ± 1 °C).

High-throughput sequencing technology has been widely employed to determine microbial diversity in food [50]. In the control samples, they exhibited the highest diversity of microorganisms among the samples (Figure 5). The genus *Lactobacillus*, while typically linked to meat spoilage, exhibits relative abundances ranging from 4.8% at 0 d to 13.7% at 12 d. Moreover, the genus *Pseudomonas* is typically found in soil and water, as well as in poultry meats [51].

There was a significant increase in the relative abundance of *Lactobacillus* in all the treatments. The genus *Lactobacillus* became the dominant bacteria, with relative abundances ranging from 17.6% and 8.1% at 0 d to 78.9% and 72.9% at 32 d in the *L. plantarum*-treated sample and *L. sakei*-treated sample, respectively. These results agreed with the results of the bacterial counts (Figure 4). This was probably due to the fact that *Lactobacillus* spp. produced inhibitory substances including organic acids, hydrogen peroxide, enzymes, and bacteriocins [21,52]. Both *L. plantarum* R2 and *L. sakei* B2 were found to inhibit the growth of *Neisseria, Fusobacterium*, *Streptococcus*, and *Brochothrix.* For the genus *Pseudomonas*, the relative abundance was clearly found. This was consistent with the results of *Pseudomonas* counts (Figure 4D).

### 3.5. Sensory Analysis

**Figure 6:**
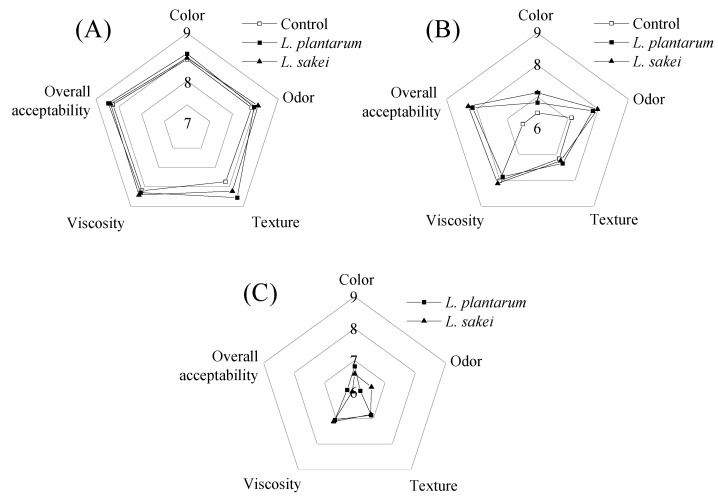
Effect of inoculating *L. plantarum* R2 and *L. sakei* B2 on the sensory quality of low-sodium sliced sausage at 0 d (**A**), 8 d (**B**) and 28 d (**C**) during refrigerated storage (5 ± 1 °C).

Sensory analysis of the chicken sausage during refrigerated storage was presented in Figure 6. At 0 d, there was no significant (*p* < 0.05) difference in color, odor, texture, viscosity, and overall acceptability among the control and the treated groups. The cooked chicken sausages treated with *L. plantarum* R2 and *L. sakei* B2 had a significantly higher score for color, odor, and overall acceptability than the non-inoculated cooked chicken sausage at 8 d, and no difference was observed between the samples containing *L. plantarum* and *L. sakei*. The control sample was given the lowest score for overall acceptability. For the texture analysis, all the samples had a similar score, which was much lower than those during the initial storage (*p* < 0.05).

The metaboli activity of some microorganisms that survived after being cooked results in meat spoilage appearing as sour, off-flavors, off-odors, milky exudates, slime production, swelling of the package through gas production, and discoloration such as greening [53]. Moreover, the multiplication of heterofermentative bacteria can lead to swelling or rupture as a consequence of the presence of CO_2_. The LABs as bioprotective cultures can fight against specific spoilage organisms in the meat products, such as *B. thermosphacta* and *Enterococcus faecalis* [49]. These microorganisms are known to negatively affect the quality of meat products [53,54]. Inoculation of the LABs as bioprotective cultures seemed not only to enhance the safety of cooked chicken sausage but also to reduce off-odors generated by fat oxidation [33,41]. The sensory evaluation demonstrated that the chicken sausage treated with *L. plantarum* and *L. sakei* was not accepted by the panelists even at 28 d. This may be due to the higher sour taste and smell of the inoculated groups.

## 4. Conclusions

This study demonstrated that the cooked chicken sausage inoculated with *L. plantarum* R2 and *L. sakei* B2 has a lower level of lipid and protein oxidation, which indicated that the LABs exerted a clear antioxidant activity. However, the antioxidant activity of the group with *L. plantarum* R2 was stronger than that with *L. sakei* B2. Moreover, the inoculation of LABs led to a significant reduction in the growth of *P. fluorescens* and *B. thermosphacta* compared to the control. And inoculation of *L. plantarum* R2 had a lower *B. thermosphacta* count compared to the inoculation of *L. sakei* B2. Regarding the abundance of meat microbial communities, the culture-independent analysis via high-throughput sequencing showed significant antimicrobial activity, which was observed in *L. plantarum* R2-inoculated cooked chicken sausage. According to the results of the sensory evaluation, *L. plantarum* R2 and *L. sakei* B2 were potential technologies for low-sodium cooked chicken sausage to improve its organoleptic characteristics. Therefore, *L. plantarum* R2 isolated from Harbin dry sausage could be considered to be an antimicrobial and antioxidant strain for applications in low-sodium meat to improve physicochemical properties, organoleptic characteristics, and food safety.

## Figures and Tables

**Table 1:** Formulations of cooked chicken sausages.

Samples	Chicken Breast (75 g)	Pork Backfat (25 g)	Polyphosphates (0.5 g)	Sugar (0.5 g)	Ice Water (10 g)	NaCl (1.75 g)	KCl (0.75 g)	Inoculation (10^7^ CFU/g)
Control	+	+	+	+	+	+	+	-
*L. plantarum*	+	+	+	+	+	+	+	*L. plantarum*
*L. sakei*	+	+	+	+	+	+	+	*L. sakei*

## Data Availability

The original contributions presented in this study are included in the article; further inquiries can be directed to the corresponding author.

## References

[1] Song J., Chen L.K., Xiong H., Ma Y., Pombo-Rodrigues S., Macgregor G.A., He F.J. (2024). Blood Pressure–Lowering Medications, Sodium Reduction, and Blood Pressure. Hypertension.

[2] Gholizadeh-Moghaddam M., Shahdadian F., Shirani F., Hadi A., Clark C.C.T., Rouhani M.H. (2023). The effect of a low versus high sodium diet on blood pressure in diabetic patients: A systematic review and meta-analysis of clinical trials. Food Sci. Nutr..

[3] WHO/FAO (World Health Organization/Food and Agriculture Organization) (2003). Diet, Nutrition and the Prevention of Chronic Diseases.

[4] Armenteros M., Aristoy M.C., Barat J.M., Toldrá F. (2009). Biochemical and sensory properties of dry-cured loins as affected by partial replacement of sodium by potassium, calcium, and magnesium. J. Agric. Food Chem..

[5] Ruusunen M., Puolanne E. (2005). Reducing sodium intake from meat products. Meat Sci..

[6] Cappuccio F.P., Beer M., Strazzullo P. (2019). Population dietary salt reduction and the risk of cardio- vascular disease. A scientific statement from the European Salt Action Network. Nutr. Metab. Cardiovas..

[7] Hu Y., Wang Q., Sun F.D., Chen Q., Xia X.F., Liu Q., Kong B.H. (2022). Role of partial replacement of NaCl by KCl combined with other components on structure and gel properties of porcine myofibrillar protein. Meat Sci..

[8] He N., Chen X.R., Li L., Wang S.Y., Lan M.J., Yuan Y., Zhang Z.H., Li T.S., Zhang X., He X. (2024). κ-carrageenan masking bitterness perception in surimi gels containing potassium chloride-based salt substitutes: Gel properties, oral processing, and sensory evaluation. Food Chem..

[9] Aleman R.S., Delarca Ruiz F., Pournaki S.K., Marcia J., Montero I., Rueda-Robles A., Borrás-Linares I., Lozano-Sánchez J. (2023). Reduced-sodium roasted chicken: Physical/technological characteristics, optimized KCl-seasoning mixture, consumer perception, liking, emotions, and purchase intent. J. Food Sci..

[10] Mizehoun-Adissoda C., Houinato D., Houehanou C., Chianea T., Dalmay F., Bigot A., Aboyans V., Preux P.-M., Bovet P., Desport J.-C. (2017). Dietary sodium and potassium intakes: Data from urban and rural areas. Nutrition.

[11] Wu H.Z., Zhang Y.Y., Li L.H., Tang J., Zhao J.Y., Ruan G.P., Zhang J.H. (2014). Influence of potassium chloride as partial substitute for sodium chloride on proteolysis and sensory properties of dry-cured meat products. Food Sci..

[12] Blesa E., Aliño M., Barat J.M., Grau R., Toldrá F., Pagán M.J. (2008). Microbiology and physico-chemical changes of dry-cured ham during the post-salting stage as affected by partial replacement of NaCl by other salts. Meat Sci..

[13] Weiss J., Gibis M., Schuh V., Salminen H. (2010). Advances in ingredient and processing systems for meat and meat products. Meat Sci..

[14] Aaslyng M.D., Vestergaard C., Koch A.G. (2014). The effect of salt reduction on sensory quality and microbial growth in hot dog sausages, bacon, ham and salami. Meat Sci..

[15] Zhang Q.S., Chen G., Shen W.X., Wang Y., Zhang W.X., Chi Y.L. (2016). Microbial safety and sensory quality of instant low-salt Chinese paocai. Food Control.

[16] Costa-Corredor A., Serra X., Arnau J., Gou P. (2009). Reduction of NaCl content in restructured dry-cured hams: Post-resting temperature and drying level effects on physicochemical and sensory parameters. Meat Sci..

[17] Doyle M.E., Glass K.A. (2010). Sodium reduction and its effect on food safety, food quality, and human health. Compr. Rev. Food Sci. Food Saf..

[18] Rodrigues I., Trindade M.A., Caramit F.R., Candoğan K., Pokhrel P.P., Barbosa-Cánovas G.V. (2016). Effect of high-pressure processing on physicochemical and microbiological properties of marinated beef with reduced sodium content. Innov. Food Sci. Emerg..

[19] Tamm A., Bolumar T., Bajovic B., Toepfl S. (2016). Salt (NaCl) reduction in cooked ham by a combined approach of high-pressure treatment and the salt replacer KCl. Innov. Food Sci. Emerg..

[20] Varsha K.K., Nampoothiri K.M. (2016). Appraisal of lactic acid bacteria as protective cultures. Food Control.

[21] Favaro L., Todorov S.D. (2017). Bacteriocinogenic lab strains for fermented meat preservation: Perspectives, challenges, and limitations. Probiotics Antimicrob..

[22] Castellano P., Belfiore C., Fadda S., Vignolo G. (2008). A review of bacteriocinogenic lactic acid bacteria used as bioprotective cultures in fresh meat produced in Argentina. Meat Sci..

[23] Slima S.B., Ktari N., Triki M., Trabelsi I., Abdeslam A., Moussa A., Moussa H., Makni I., Herrero A.M., Jiménez-Colmenero F. (2018). Effects of probiotic strains, *Lactobacillus plantarum* TN8 and *Pediococcus acidilactici* on microbiological and physico-chemical characteristics of beef sausages. LWT-Food Sci. Technol..

[24] Comi G., Tirloni E., Andyanto D., Manzano M., Lacumin L. (2015). Use of bio-protective cultures to improve the shelf-life and the sensorial characteristics of commercial hamburgers. LWT-Food Sci. Technol..

[25] Hu P., Xu X.L., Zhou G.H., Han Y.Q., Xu B.C., Liu G.C. (2008). Study of the Lactobacillus sakei protective effect towards spoilage bacteria in vacuum packed cooked ham analyzed by PCR–DGGE. Meat Sci..

[26] Liu D.C., Tsan R.T., Lin Y.C., Jan S.S., Tan F.J. (2009). Effect of various levels of rosemary or Chinese mahogany on the quality of fresh chicken sausage during refrigerated storage. Food Chem..

[27] Jayawardana B.C., Liyanage R., Lalantha N., Iddamalgoda S., Weththasinghe P. (2015). Antioxidant and antimicrobial activity of drumstick (*Moringa oleifera* ) leaves in herbal chicken sausages. LWT-Food Sci. Technol..

[28] Devatkal S.K., Naveena B.M. (2010). Effect of salt, kinnow and pomegranate fruit by-product powders on color and oxidative stability of raw ground goat meat during refrigerated storage. Meat Sci..

[29] Berardo A., Maere H.D., Stavropoulou D.A., Rysman T., Leroy F., De Smet S. (2016). Effect of sodium ascorbate and sodium nitrite on protein and lipid oxidation in dry fermented sausages. Meat Sci..

[30] Xiao Y.Q., Li P.J., Zhou Y., Ma F., Chen C.G. (2018). Effect of inoculating *Lactobacillus pentosus* R3 on N-nitrosamines and bacterial communities in dry fermented sausages. Food Control.

[31] (2008). Criterion for Sensory Evaluation of Meat and Meat Products.

[32] Sallam K.I., Ishioroshi M., Samejima K. (2004). Antioxidant and antimicrobial effects of garlic in chicken sausage. LWT-Food Sci. Technol..

[33] Geeta, Yadav A.S. (2017). Antioxidant and antimicrobial profile of chicken sausages prepared after fermentation of minced chicken meat with *Lactobacillus plantarum* and with additional dextrose and starch. LWT-Food Sci. Technol..

[34] Li S.Y., Zhao Y.J., Zhang L., Zhang X., Huang L., Li D., Niu C.H., Yang Z.N., Wang Q. (2012). Antioxidant activity of *Lactobacillus plantarum* strains isolated from traditional Chinese fermented foods. Food Chem..

[35] Sun Q.X., Chen Q., Li F.F., Zheng D.M., Kong B.H. (2016). Biogenic amine inhibition and quality protection of Harbin dry sausages by inoculation with *Staphylococcus xylosus* and *Lactobacillus plantarum*. Food Control.

[36] Tang W., Xing Z.Q., Li C., Wang J.J., Wang Y.P. (2017). Molecular mechanisms and in vitro antioxidant effects of *Lactobacillus plantarum* ma2. Food Chemi..

[37] Han D., Deng S.Y., Wang H., Huang F., Fauconnier M.L., Li H., Zheng J., Meng L.C., Zhang C.H., Li X. (2023). Lipid oxidation and flavor changes in saturated and unsaturated fat fractions from chicken fat during a thermal process. Food Funct..

[38] Horita C.N., Messias M.C., Morgano M.A., Hayakawa F.M., Pollonio M.A.R. (2014). Textural, microstructural and sensory properties of reduced sodium frankfurter sausages containing mechanically deboned poultry meat and blends of chloride salts. Food Res. Int..

[39] Ankaiah D., Mitra S., Srivastava D., Sivagnanavelmurugan M., Ayyanna R., Jha N., Venkatesan A. (2021). Probiotic characterization of bacterial strains from fermented South Indian tomato pickle and country chicken intestine having antioxidative and antiproliferative activities. J. Appl. Microbiol..

[40] Park W., Kim J.H., Ju M.G., Hong G.E., Yeon S.J., Han G.S., Lee C.H. (2017). Enhancing quality characteristics of salami sausages formulated with whole buckwheat flour during storage. J. Food Sci. Technol..

[41] Sun F.D., Kong B.H., Chen Q., Han Q., Diao X.P. (2017). *N*-nitrosoamine inhibition and quality preservation of Harbin dry sausages by inoculated with *Lactobacillus pentosus*, *Lactobacillus curvatus*, and *Lactobacillus sake*. Food Control.

[42] Xu M.F., Sun M.F., Lu C.R., Han Y.T., Yao X., Niu X.Y., Xu M.J., Zhu Q. (2021). Influence of epicatechin on oxidation-induced physicochemical and digestibility changes in porcine myofibrillar proteins during refrigerated storage. J. Sci. Food Agr..

[43] Karabacak S., Bozkurt H. (2008). Effects of *Urtica dioica* and *Hibiscus sabdariffa* on the quality and safety of sucuk (Turkish dryfermented sausage). Meat Sci..

[44] Tsafrakidou P., Sameli N., Bosnea L., Chorianopoulos N., Samelis J. (2021). Assessment of the spoilage microbiota in minced free-range chicken meat during storage at 4C in retail modified atmosphere packages. Food Microbiol..

[45] Kreyenschmidt J., Hübner A., Beierle E., Chonsch L., Scherer A., Petersen B. (2010). Determination of the shelf life of sliced cooked ham based on the growth of lactic acid bacteria in different steps of the chain. J. Appl. Microbiol..

[46] Andritsos N.D., Mataragas M., Mavrou E., Stamatiou A., Drosinos E.H. (2012). The microbiological condition of minced pork prepared at retail stores in athens, greece. Meat Sci..

[47] Papadopoulou O.S., Doulgeraki A.I., Botta C., Cocolin L., Nychas G.J.E. (2012). Genotypic characterization of *Brochothrix thermosphacta* isolated during storage of minced pork under aerobic or modified atmosphere packaging conditions. Meat Sci..

[48] Russo F., Ecolini D., Mauriello G., Villani F. (2006). Behaviour of *Brochothrix thermosphacta* in presence of another meat spoilage microbial groups. Food Microbiol..

[49] Spanu C., Piras F., Mocci A.M., Nieddu G., De Santis E.P.L., Scarano C. (2018). Use of *Carnobacterium spp* protective culture in MAP packed Ricotta fresca cheese to control *Pseudomonas* spp. Food Microbiol..

[50] Shokralla S., Spall J.L., Gibson J.F., Hajibabaei M. (2012). Next-generation sequencing technologies for environmental DNA research. Mol. Ecol..

[51] Snyder A.B., Martin N., Wiedmann M. (2024). Microbial food spoilage: Impact, causative agents and control strategies. Nat. Rev. Microbiol..

[52] Ammor M.S., Mayo B. (2007). Selection criteria for lactic acid bacteria to be used as functional starter cultures in dry sausage production: An update. Meat Sci..

[53] Wang G.Y., Wang H.H., Han Y.W., Xing T., Ye K.P., Xu X.L., Zhou G.H. (2017). Evaluation of the spoilage potential of bacteria isolated from chilled chicken in vitro, and in situ. Food Microbiol..

[54] Casaburi A., Martino V.D., Ercolini D., Parente E., Villani F. (2015). Antimicrobial activity of *myrtus communis L.* water-ethanol extract against meat spoilage strains of *Brochothrix thermosphacta*, and *pseudomonas fragi*, in vitro and in meat. Ann. Microbiol..

